# Bone defects are repaired by enhanced osteogenic activity of the induced membrane: a case report and literature review

**DOI:** 10.1186/s12891-021-04317-2

**Published:** 2021-05-15

**Authors:** Ye Lu, Jian Wang, Ying Yang, Qudong Yin

**Affiliations:** 1 Department of Orthopaedics, Wuxi No. 9 People’s Hospital Affiliated to Suzhou University, Liangxi Road No. 999, Wuxi, 214062 Jiangsu China; 2 Department of Radiology, Wuxi No. 9 People’s Hospital Affiliated to Suzhou University, Liangxi Road No. 999, Wuxi, 214062 Jiangsu China

**Keywords:** Induced membrane, Bone defect, Case report, Osteogenesis, Repair surgery

## Abstract

**Background:**

The induced membrane technique (IMT) is an effective strategy to repair bone defects and involves a two-stage set of surgical procedures. Although the IM has osteogenic activity, bone grafting is necessary in standard IMT. Bone defects repaired completely by osteogenic activity of the IM alone without bone grafts are rare.

**Case presentation:**

We present a case of infected fractures and bone defects of the ulna and radius treated with IMT. After the first stage using polymethylmethacrylate (PMMA) beads, X-rays showed that new callus developed after 2 to 4 months, and the defects were repaired completely by 5 months. We also present a literature review on spontaneous osteogenesis of the IM in patients.

**Conclusions:**

We present a case of infected ulnar and radial bone defects that healed by 5 months after the first stage of the IMT using a PMMA spacer. This finding suggests that local associated inflammatory reactions and bone tissue might enhance the osteogenic activity of the IM, causing spontaneous healing of bone defects. This appears to be the first such case reported in the literature.

## Background

Bone defects in the extremities caused by severe trauma, infection and tumor resection are common in clinical practice. Bone defects greater than two cm cannot heal spontaneously, and reconstruction surgery is necessary [1, 2]. The induced membrane technique (IMT) is a recently developed effective strategy to repair bone defects [3–5]. Standard IMT includes a two-stage set of surgical procedures. The first stage involves insertion of a polymethylmethacrylate (PMMA) cement spacer after radical debridement of bone and soft tissue. A membrane around the spacer formed as a foreign body reaction is known as an induced membrane (IM). The second stage is bone grafting within the membrane after removal of the cement spacer [4, 5]. The mechanisms of IMT mainly include the following: (i) the IM acts as a barrier to prevent the soft tissue from growing into the fracture site, and plays a role as a wrapping device to prevent loosening and absorption of the bone graft; (ii) the IM secretes osteogenic factors and contains mesenchymal stem cells (MSCs), which express bone morphogenetic protein-2 (BMP-2), vascular endothelial growth factor (VEGF) and transforming growth factor-β (TGF-β); (iii) the surface of the IM has rich micro-vessels to increase the blood supply [1, 3]. Therefore, the IM has intrinsic osteogenic activity [6]. However, bone defects repaired completely by intrinsic osteogenic activity of the IM alone are rare in clinical practice. We report a case of infected fractures with ulnar and radial bone defects that were repaired completely by osteogenic activity of the IM and review the literature on spontaneous osteogenesis of the IM.

## Case presentation

**FIG. 1:**
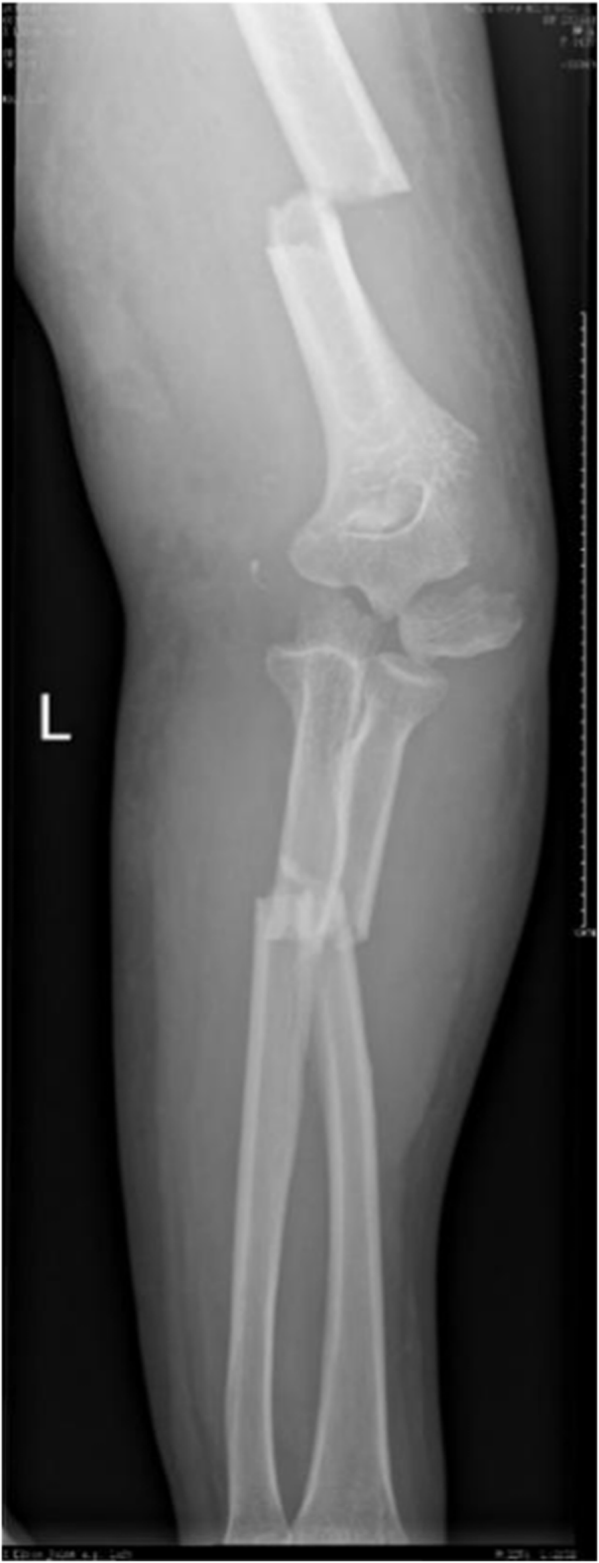
AP radiograph of fractures showing the left humerus, ulna and radius on admission

**FIG. 2:**
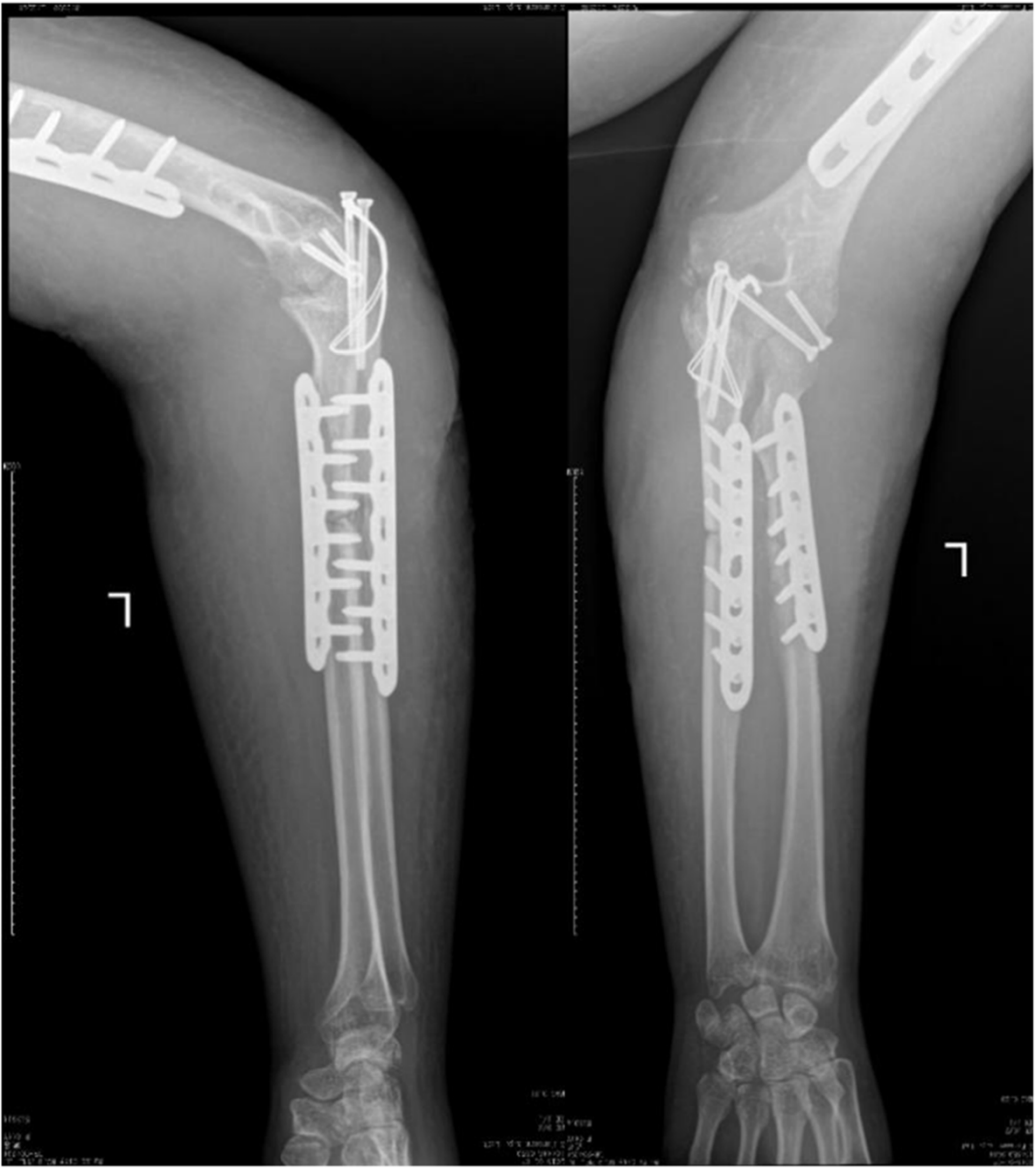
Lateral radiograph showing fractures of the left humerus, ulna and radius after open reduction and internal fixation using plates and screws

**FIG. 3:**
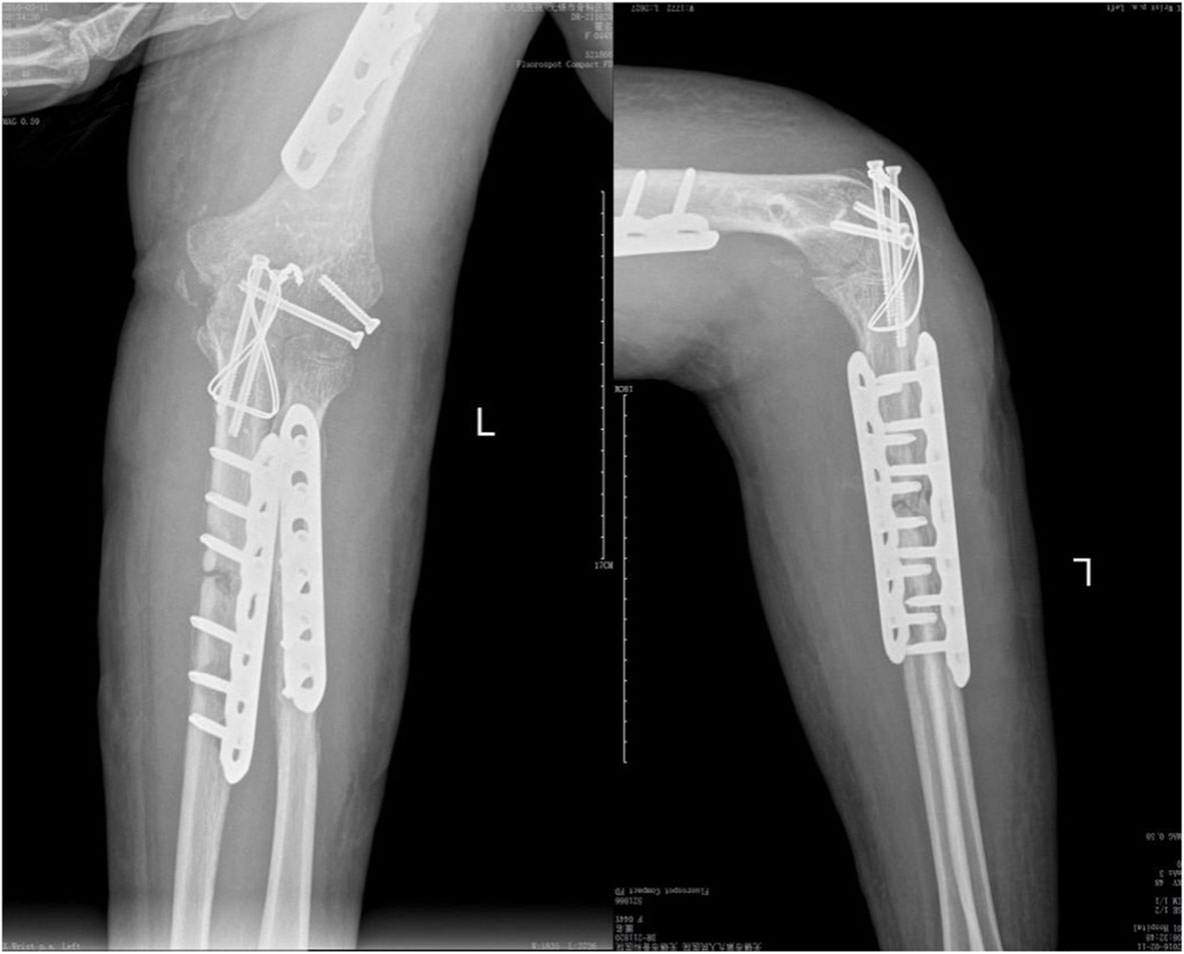
AP and lateral radiographs showing obvious bone resorption and a slight periosteal reaction

**FIG. 4:**
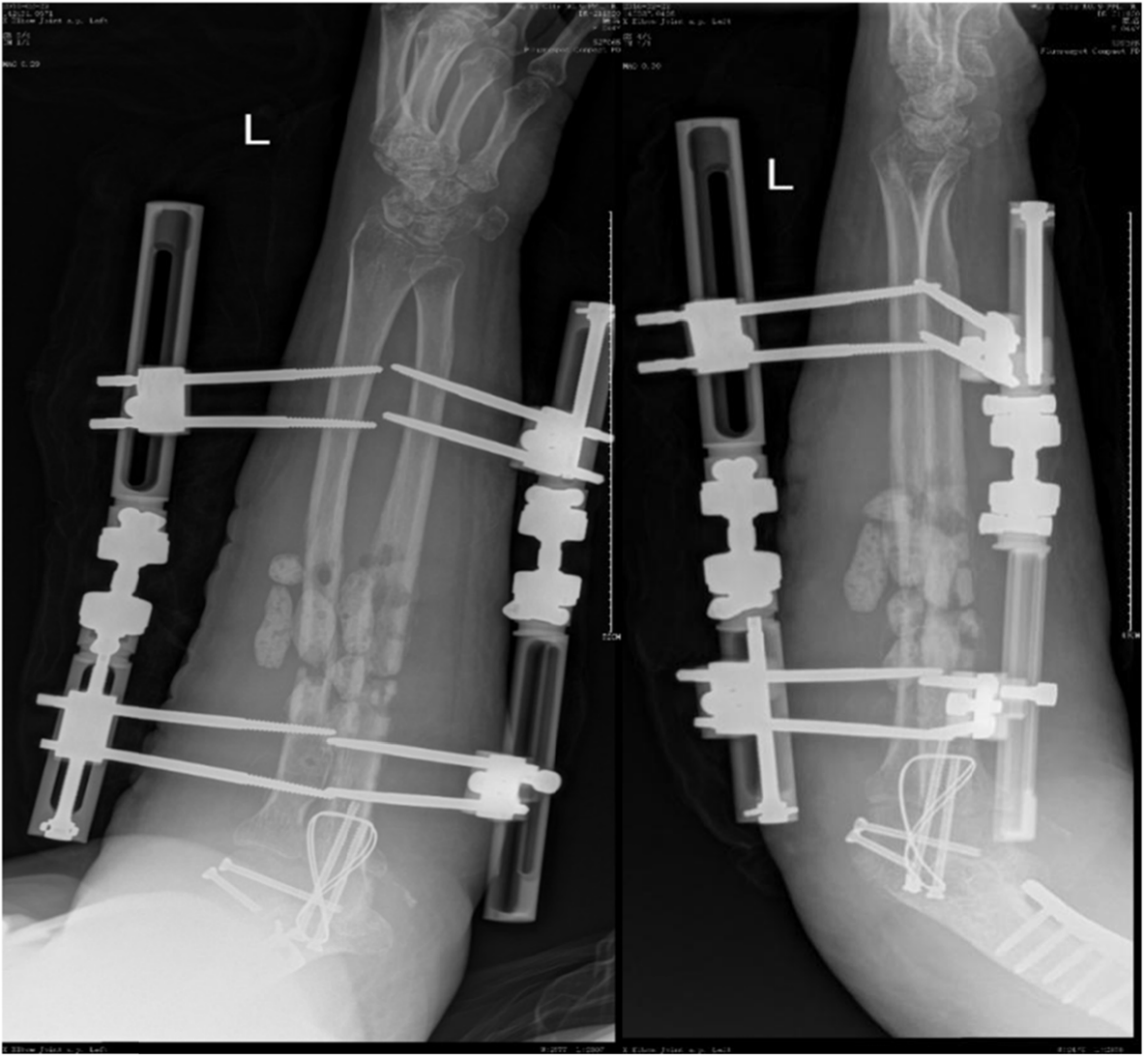
AP and lateral radiographs showing bone cement beads inserted into and around the defects and the bone defect fixed externally after the first stage of the IMT for the infected ulnar and radial fractures

**FIG. 5:**
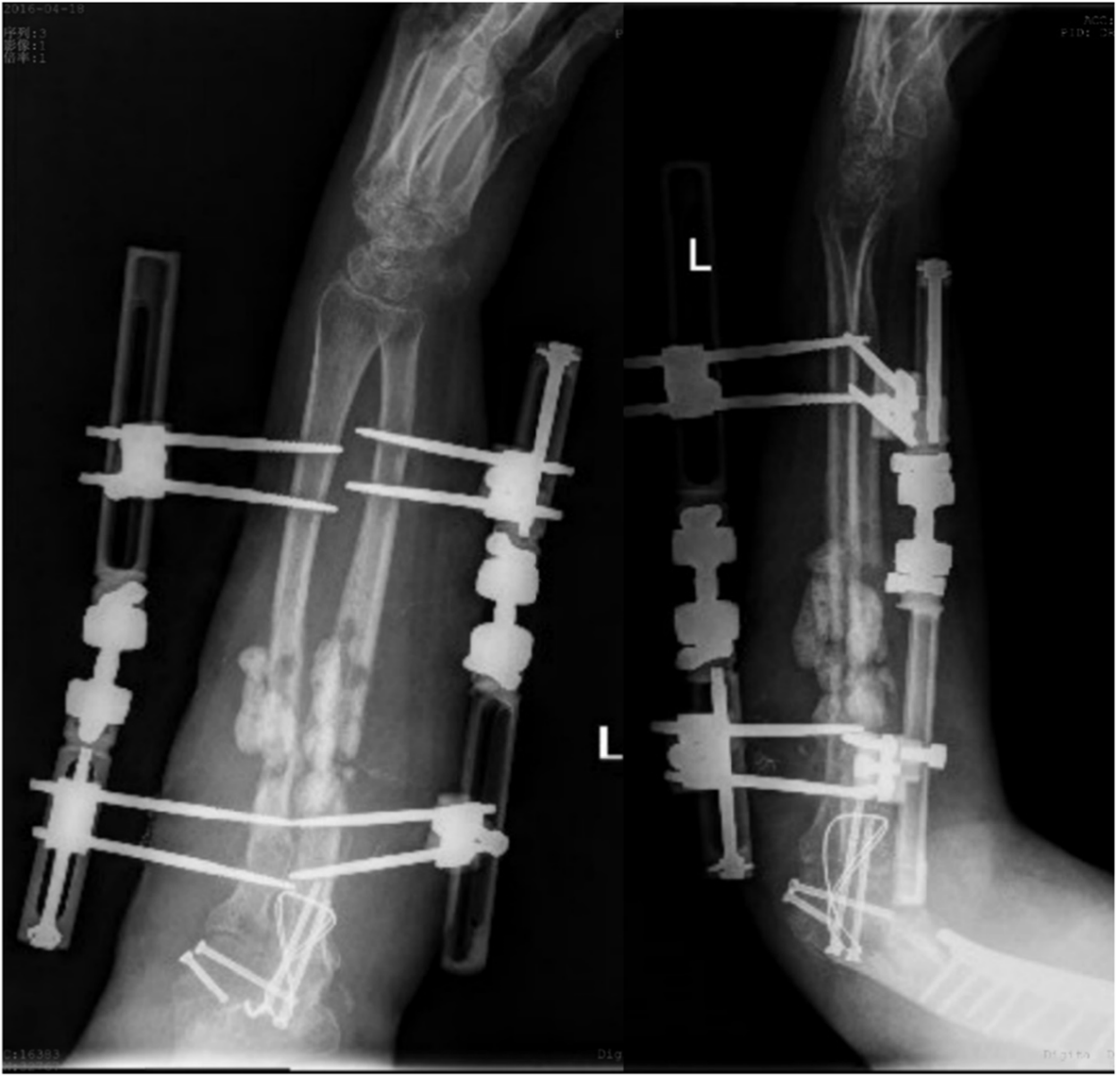
AP and lateral radiographs showing less new callus developed 2 months after the first stage procedure of the IMT

**FIG. 6:**
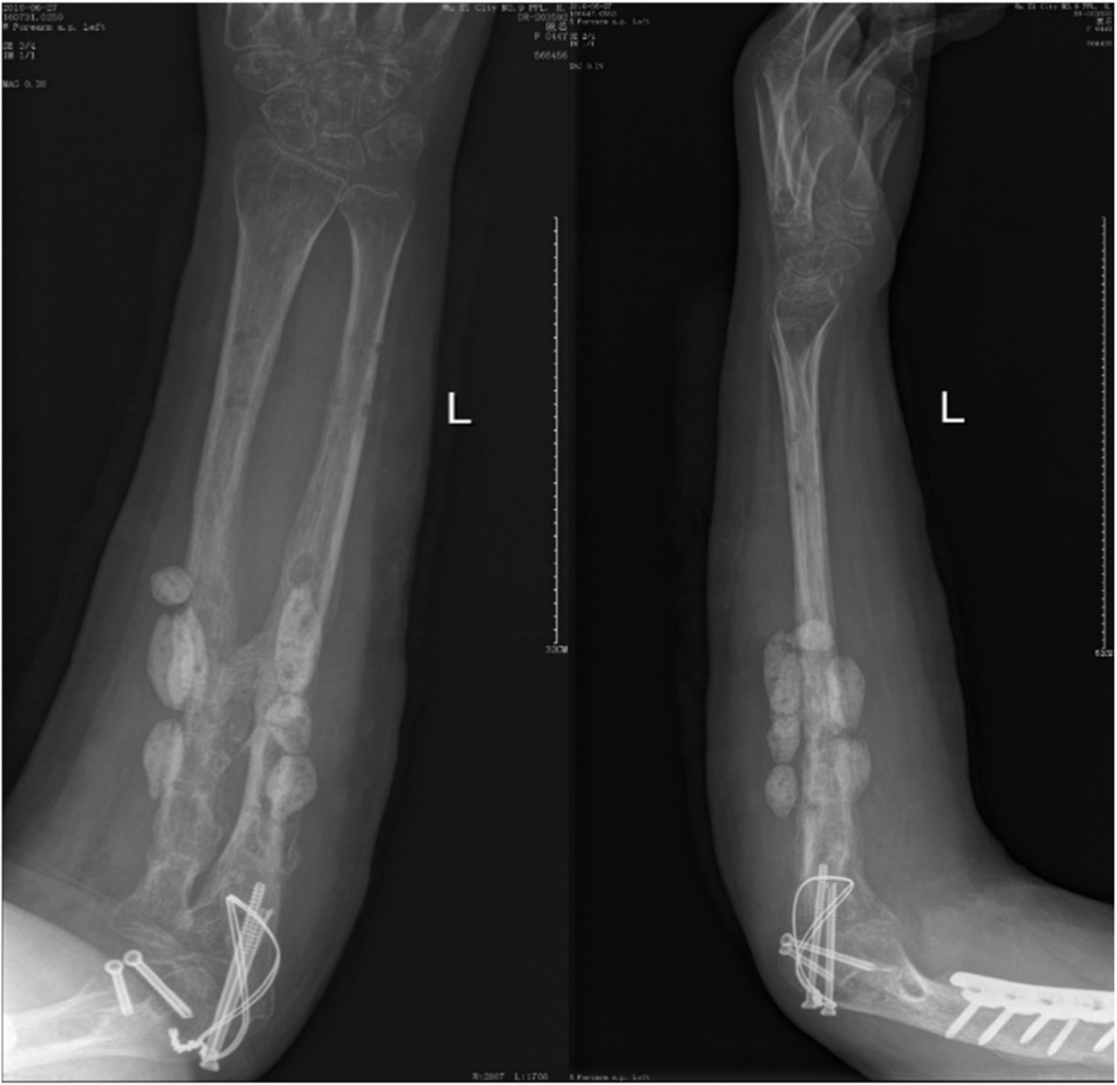
AP and lateral radiographs showing new callus continuing to develop and connecting the defects 4 months after insertion of PMMA

**FIG. 7:**
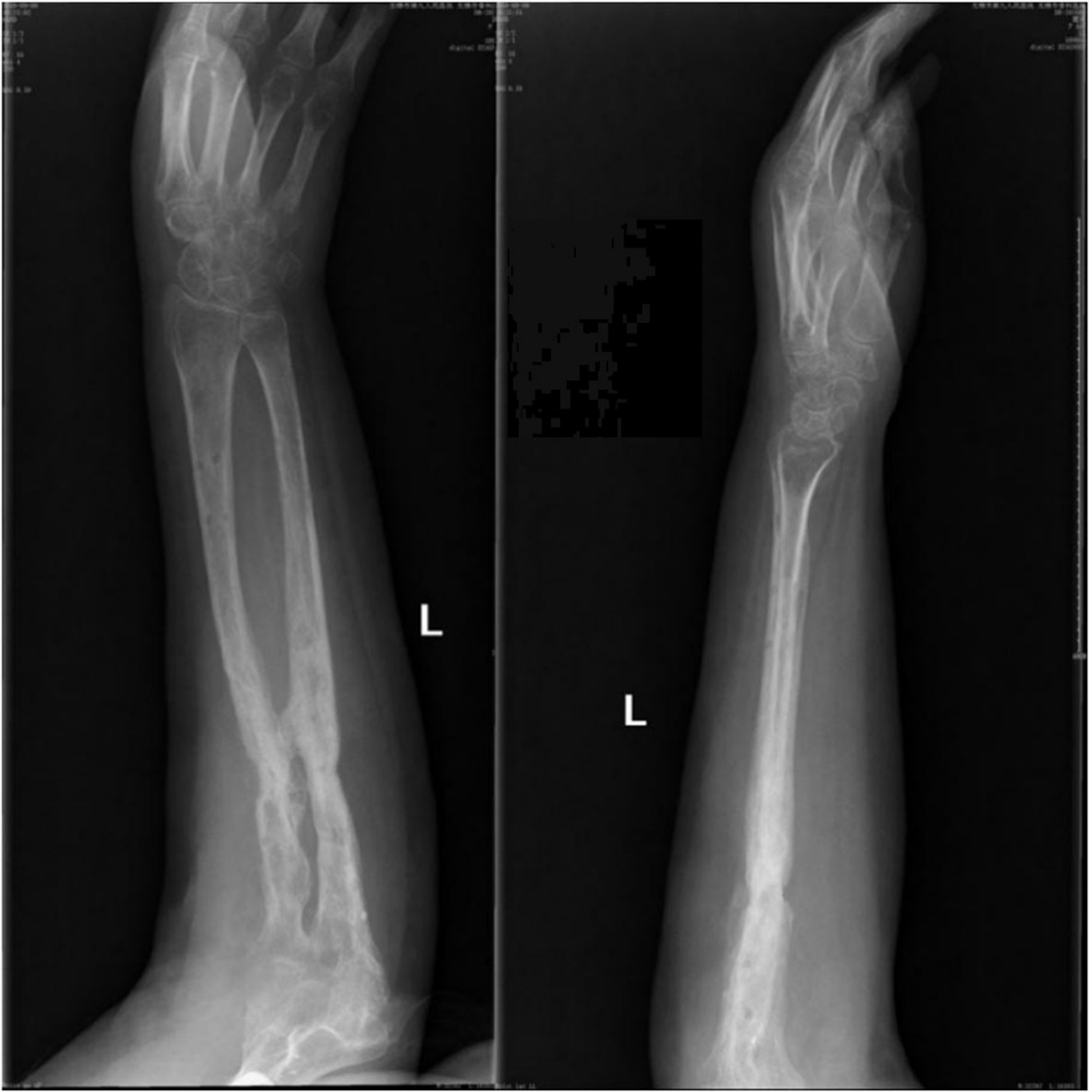
AP and lateral radiographs showing clinical healing of the fractures and defects and removal of spacers 6 months after insertion of PMMA

A previously fit and healthy 44-year-old female with no history of hypertrophic ossifications was admitted with a complaint of pain and limited movement of the left upper limb following an accident in which her arm was entangled and twisted injury in a machine. Physical examinations and X-rays demonstrated that the patient had fractures of the left humerus, ulna and radius (AO classification: 12-A3, 13-B1, 22-A3) with a forearm skin contusion (Fig. 1). Open reduction and definitive stabilization using plates and screws of the fractures of the left humerus, ulna and radius were performed immediately on admission (Fig. 2). She was discharged after the incisions healed. Six weeks following internal fixation, her left forearm became red, swollen and painful. A small hole was made, and pus began to exude. *Pseudomonas aeruginosa* was cultured from the secretion. X-rays showed obvious bone resorption and slight periosteal reactions on the left ulna and radius (Fig. 3). A deep incision infection after internal fixation of the ulna and radius fractures was confirmed. Because conservative treatment is invalid, surgical intervention was required. Following radical debridement including removal of the hardware, 0.7 cm segmental defects of the fracture ends, and 4–5 cm partial defects of the ulna and radius were noted. External fixators were used for fixation and maintaining the length of the ulna and radius, then PMMA bone cement beads loaded with vancomycin (40:4) were inserted into and around the defects (Fig. 4). Cefmetazole was administered intravenously for 2 weeks, followed by oral rifampin for 4 weeks. The wound healed uneventfully, and she was encouraged to perform early postoperative rehabilitation activities. Follow-up X-rays two and 4 months after the first stage displayed a continuing process of new bone regeneration in the bone defects (Figs. 5 and 6). The ulnar and radial fractures and defects were healed by clinical evaluation by 4.5 months, and the external fixators were removed in the clinic at 5.5 months. The second stage of the IMT (bone grafting) was not necessary. The bone cement spacers were removed 6 months after filling with PMMA (Fig. 7). Intraoperative exploration revealed that parts of the spacers were wrapped by new callus. No additional surgeries were between the two surgeries. The humeral fracture healed by 3 months postoperatively. The patient recovered the normal length of her humerus, ulna and radius, as well as most of the function of her left upper limb. One year postoperatively, there was no recurrence of infection, her hand grip strength recovered to 80% of the contralateral side, her range of motion: wrist flexion recovered to 56°, wrist extension recovered to 53°, pronation of the forearm recovered to 69°, supination recovered to 73°, elbow flexion recovered to 95°, and elbow extension recovered to 0°.

## Method of literature review

Studies were retrieved using the terms “bone defect”, “IMT” or “Masquelet technique”, “osteogenesis” or “bone formation” and “bone healing” from PubMed and Web of Science databases between 2000 and 2020 to collect cases of bone defects repaired using the IMT or Masquelet technique using PMMA as a spacer, in which new bone formed without bone graft.

## Retrieval results

There were only two case reports of bone defects repaired by IMT, in which new bone was found with detailed clinical and follow-up data. One of these patients was a 42-year-old male with severe femoral fractures and defects accompanied by traumatic brain injury (TBI). The interval from filling with PMMA to clinical healing was 48 days [7]. The other was a 7-year-old male boy with mandibular Ewing’s sarcoma treated by a combination of neoadjuvant chemotherapy, resection surgery, and adjuvant radiochemotherapy. A macroplate and a PMMA cement spacer were used for the reconstruction. The spacer was removed after 17 days. X-ray films 2 months postoperatively showed that a small amount of new bone had formed along the macroplate; however, bone grafting was performed in a second stage of surgery [8].

## Discussion

In 2009, Klaue et al. [6] first found in an animal model that the IM is spontaneously osteogenic. They designed a reproducible animal model producing a stable 3 cm mid-diaphyseal bone resection and periosteal defects on sheep femurs and created a foreign-body membrane. After removal of the spacer, radiographs in the group without bone grafts at 16–18 weeks demonstrated that a small amount of woven bone formed at the proximal junction of the bone end and the IM. However, the spontaneous osteogenesis of the IM was weak. No constant osteogenesis was observed in other studies, so it did not attract attention.

The rapid callus formation in the case of femoral fractures and defects accompanying TBI reported by Hotchen et al. [7] was attributed to the dual effects of the TBI and the IM, especially the TBI. The exact mechanisms of fracture that accompany TBI to increase callus formation are not yet completely understood. Potential mechanisms may include: (i) Bone fracture triggers the release of cytokines including tumor necrosis factor-α (TNF-α), interleukin-1 (IL-1) and interleukin-6 (IL-6) in both the serum and the cerebrospinal fluid that attract MSCs to the fracture site and generate an osteogenic effect [7, 8] . (ii) The enhanced inflammatory response caused by TBI creates a receptive environment for the occurrence of heterotopic ossification (HO), and the upregulation of some inflammatory factors in the body fluids accelerates bone formation. (iii) Leptin is increased in the circulation following TBI and is also released in the stress response to cytokines and hormonal factors. Leptin is positively correlated with the amount of callus formation at a fracture site [7, 9]. (iv) Patients who suffer from TBI are typically in a coma and require mechanical ventilation. The combination of these factors promotes the incidence of HO and rapid bone formation in TBI patients.

A 7-year-old boy with sarcoma resection of the mandibular branch was reported by Sorin et al. [8]. Only a small amount of new bone was found in the boy and this was attributed to the osteogenic activity of the IM and younger age.

The case presented in the current study involved a situation in which the infected bone defects healed spontaneously by osteogenic activity of the IM alone. This case differed from that of Hotchen et al. [7] which was based on the presence of TBI, and from that of Sorin et al. [8] based on the amount of osteogenesis. The possible reasons for spontaneous healing of the bone defects are: (i) the local inflammatory responses increase the release of inflammatory factors, which increase the thickness of the IM and the level of vascularization as well as recruitment of pro-osteogenic cytokines [10–12] and (ii) some fracture fragments or periosteum are present near the cement spacer, which can provide seed cells and enhance the osteogenic activity of the IM [13–15].

Catros et al. [14] conducted an in vivo experiment to investigate the osteoinductive properties of the IM on a macroporous hydroxyapatite-tricalcium phosphate (HA-TCP) scaffold in a non-osseous subcutaneous site in rabbits. The results showed that the IM placed in a non-osseous site had no osteoinductive properties. In this current case, callus growth was observed only on the side near the bone defects, but not on the side near the subcutaneous tissue, consistent with the results of Catros et al. [14] and Klaue et al. [6]. In view of the local associated inflammatory responses, fracture fragments or periosteum around the cement spacer may improve the osteogenic activity of the IM, causing spontaneous healing of the bone defects. Therefore, we assume that filling with PMMA beads as a spacer for partial bone defects or adding a small amount of bone graft for segmental bone defects around the beads may stimulate spontaneous osteogenesis. Of course, this hypothesis needs to be tested experimentally.

It is a slow process to completely repair bone defects by the osteogenic activity of IM alone. This is especially true in the long bones of the lower limb because it may lead to stiffness of adjacent joints and other sequelae; it is not a preferred method at present. The infection of the incision after internal fixation of the ulnar and radial fractures was related to improper surgery for a complex severe injury of the upper extremity (floating elbow) combined with a forearm skin contusion, or to improper aseptic conditions. The lesson of this case is that for a complex severe injury with a skin contusion, multiple site incisions, especially large incisions, for open reduction and internal fixation should not be performed emergently at the same time.

## Conclusions

We present a case of infected bone defects of the ulnar and radial bone. After the first stage of the IMT using PMMA beads, X-rays showed that new callus developed and the defects healed by 5 months. This finding most likely can be attributed to local associated inflammatory reactions and bone tissue, which enhance the osteogenic activity of the IM, causing spontaneous healing of the bone defects. This case differed from those reported in the literature. It appears to be the first such case reported in the literature.

## Data Availability

This is a case report of a single patien, the imaging data are presented within the manuscript. The original reports, laboratory studies, imaging studies and outpatient clinic records are retained as per normal procedure within the medical records of our institution.

## Abbreviations

IMT: Induced membrane technique
IM: Induced membrane
PMMA: Polymethylmethacrylate
MSC: Mesenchymal stem cell
TBI: Traumatic brain injury
HO: Heterotopic ossification
